# Supplementary material for: Influence of Culture Conditions on the Bioreduction of Organic Acids to Alcohols by Thermoanaerobacter pseudoethanolicus
Source: Microorganisms. 2021 Jan 12;9(1):162. doi: 10.3390/microorganisms9010162 (PMC7828175; doi:10.3390/microorganisms9010162)
Supplement: Supplementary file 1 [file microorganisms-09-00162-s001.pdf]

Supplemental Material

Influence of Culture Conditions on the Bioreduction of Organic Acids to Alcohols by *Thermoanaerobacter pseudoethanolicus*

Sean Michael Scully[1], Aaron Brown[2], Yannick Mueller-Hilger[1], Andrew B. Ross[2], Jóhann Örlygsson[1*]

[1]Faculty of Natural Resource Science, University of Akureyri, Borgir v. Nordurslod, 600 Akureyri, Iceland.
[2]School of Chemical and Process Engineering, University of Leeds, Leeds, LS2 9JT, United Kingdom.
Corresponding author E-mail; jorlygs@unak.is

Supplemental Figure 1 - Time-course studies of yeast extract (control) fermentation by *T. pseudoethanolicus*. Values represent the average of triplicate fermentations with standard deviation presented as error bars.

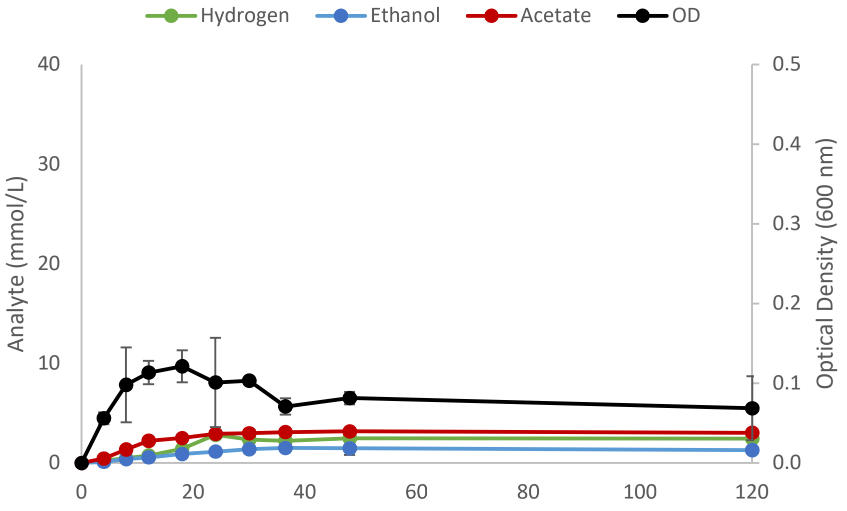

Supplementary Figure 2 – Influence of liquid-gas phase ratio on end product formation of T. pseudoethanolicus grown on glucose (20 mM) supplemented a carboxylic acid (20 mM). Values represent the average of triplicate measurements ± standard deviation.

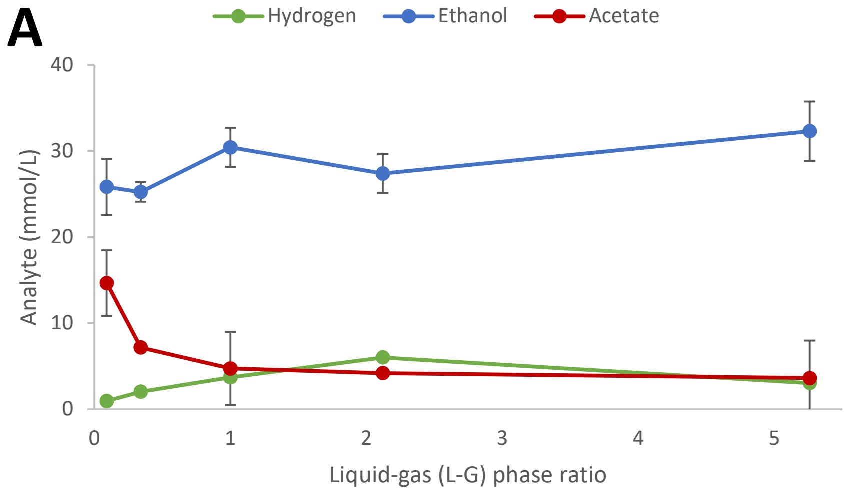

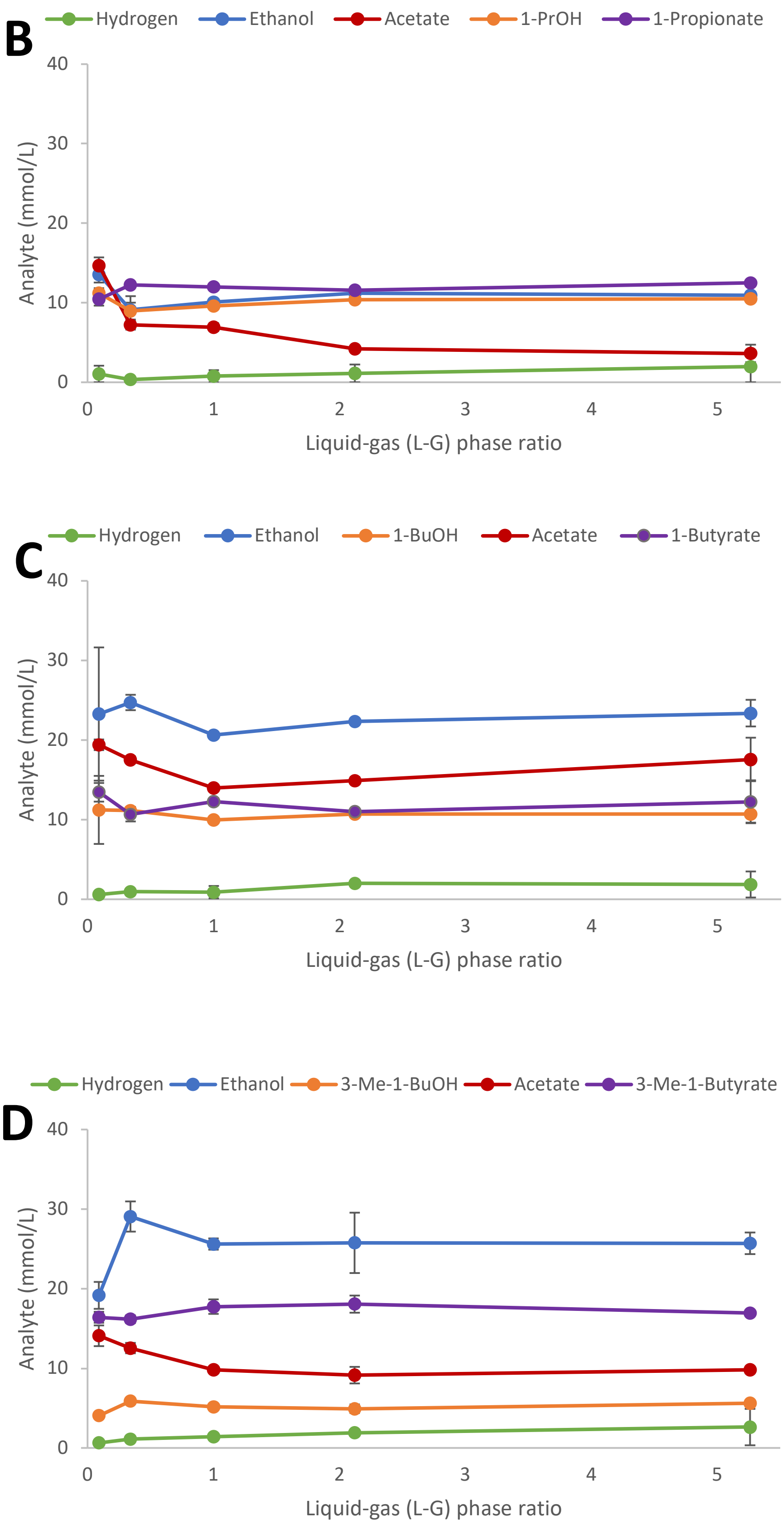
B
Hydrogen
Ethanol
Acetate
1-PrOH
1-Propionate
Analyte (mmol/L)
0
10
20
30
40
0
1
2
3
4
5
Liquid-gas (L-G) phase ratio
C
Hydrogen
Ethanol
1-BuOH
Acetate
1-Butyrate
Analyte (mmol/L)
0
10
20
30
40
0
1
2
3
4
5
Liquid-gas (L-G) phase ratio
D
Hydrogen
Ethanol
3-Me-1-BuOH
Acetate
3-Me-1-Butyrate
Analyte (mmol/L)
0
10
20
30
40
0
1
2
3
4
5
Liquid-gas (L-G) phase ratio

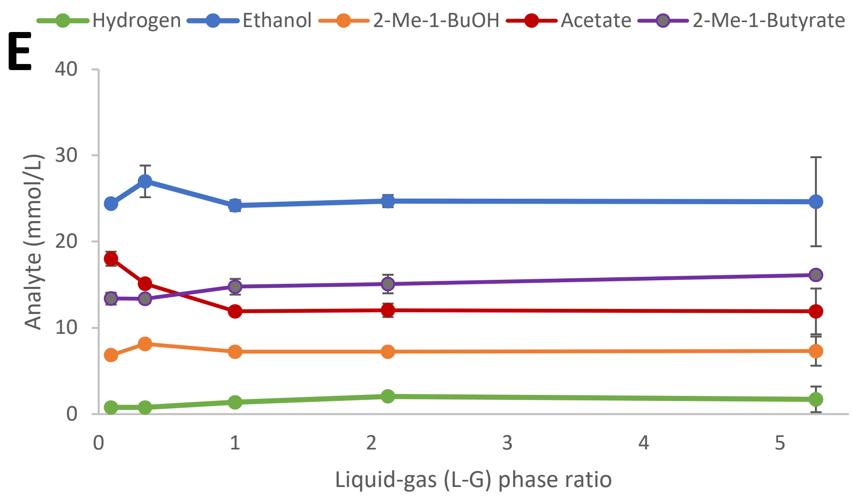
E
Hydrogen
Ethanol
2-Me-1-BuOH
Acetate
2-Me-1-Butyrate
Analyte (mmol/L)
40
30
20
10
0
0
1
2
3
4
5
Liquid-gas (L-G) phase ratio
